# The burden of amyloid light chain amyloidosis on health-related quality of life

**DOI:** 10.1186/s13023-016-0564-2

**Published:** 2017-01-19

**Authors:** Martha Bayliss, Kristen L. McCausland, Spencer D. Guthrie, Michelle K. White

**Affiliations:** 1 Optum, 24 Albion Road, Lincoln, RI 02865 USA; 2 Prothena Biosciences Inc, South San Francisco, CA USA

**Keywords:** Amyloidosis, Burden, Quality of life, Rare disease, SF-36

## Abstract

**Background:**

Light chain (AL) amyloidosis is a rare disease characterized by misfolded amyloid protein deposits in tissues and vital organs, and little is known about the burden of AL amyloidosis on health-related quality of life. This study aimed to quantify the burden of AL amyloidosis in terms of health-related quality of life in a diverse, community-based sample of AL amyloidosis patients.

**Results:**

The SF-36v2® Health Survey (SF-36v2), a widely used generic measure of health-related quality of life (using physical and mental summary scales and subscales assessing eight aspects of functioning and well-being), was administered as an online survey of AL amyloidosis patients with AL amyloidosis (ClinicalTrials.gov, NCT02574676; *n* = 341). Compared with adjusted general population sample norms, health-related quality of life of AL amyloidosis patients was significantly worse across all SF-36v2 scales and summary measures based on analysis of variance (*p* < 0.05 for all). The largest decrement in AL amyloidosis patients was related to General Health (Δ = 9.7; *p* < 0.001). With the exception of Bodily Pain and Mental Health, differences were also clinically meaningful based on established clinically minimal important differences. The burden of AL amyloidosis overall and in key subgroups tended to be greater on physical health than on mental health. Stratified analyses indicated additional burden among patients with recently diagnosed disease and those with cardiac involvement than among their respective counterparts.

**Conclusion:**

Understanding the burden of AL amyloidosis highlights the unmet need for treatment, helps physicians identify ancillary treatments and services geared towards improving patients’ functioning, well-being, and overall health-related quality of life. These findings also help to support the use of health-related quality of life end points as important outcome measures in current and future treatment studies.

**Trial registration:**

ClinicalTrials.gov, NCT02574676. Registered October 5, 2015.

## Background

Systemic amyloidoses are a group of rare diseases characterized by abnormally folded protein (amyloid) deposits in body tissue and organs. Accumulation of these insoluble amyloid deposits can lead to organ toxicity, irreversible organ damage, dysfunction, and death [1]. Amyloidosis subtypes are defined by their fibril composition and precursor proteins. In amyloid light chain (AL) amyloidosis, the amyloid deposits are created by immunoglobulin light chain proteins produced by abnormal monoclonal plasma cells [2]. The estimated incidence of AL amyloidosis is 8 to 12 cases per million person-years [3, 4].

AL amyloidosis is a complex disease with a variety of clinical manifestations, nonspecific symptoms that are associated with a wide range of diseases, and a high-case fatality rate-factors that can contribute to challenges in early diagnosis [5]. Symptoms and complications depend on the number and types of organ systems involved and the duration of time between symptom onset and treatment. Although estimates vary, approximately 70% of patients have cardiac involvement and 68% have multiple organ involvement at diagnosis [6]. Damage to multiple organs and cardiac impairment are predictive of decreased survival rates and poor disease outcomes; the most common cause of death is cardiac complication [7].

Prognosis for patients with AL amyloidosis is improved by early diagnosis and treatment. No treatments have been approved by either the United States Food and Drug Administration (FDA) or the European Medicines Agency (EMA), leaving substantial unmet need for patients. Typical treatments, which are adapted from regimens used to treat multiple myeloma, include chemotherapy, stem cell transplantation (SCT), and immunomodulatory drugs. These treatments target plasma cells and aim to reduce the production of amyloid-forming light chains. Existing regimens can be associated with significant tolerability problems, including treatment-emergent symptoms [8, 9].

Understanding the patient experience, including physical and mental aspects of health-related quality of life (HRQoL), can help to characterize burden of disease. Qualitative research indicates that patients with AL amyloidosis experience substantial burden that can lead to impairment in daily functioning [10, 11]. Furthermore, AL amyloidosis can lead to anxiety, frustration, and depression as patients grapple with the gravity and rarity of their condition [10, 12]. Although limited, quantitative studies provide evidence of deficits in both physical and mental aspects of HRQoL [13, 14].

Given the absence of a disease-specific measure of HRQoL for AL amyloidosis and the disease heterogeneity, a general health status measure, such as the SF-36v2 Health Survey® (SF-36v2), is well suited for assessing HRQoL in this population [15]. The SF-36v2 is a widely used generic measure of HRQoL and the most common patient-reported outcome end point in clinical trials [16–18]. Generic measures of HRQoL allow for comparisons with the general population and other disease populations, which can provide context for rare diseases [19, 20].

Studies examining the impact of AL amyloidosis on HRQoL, particularly among patient subgroups, are scant. Accordingly, this study used the SF-36v2 to compare the HRQoL burden of AL amyloidosis patients and two key subgroups hypothesized to have greater disease severity with that observed in the US general population (GP).

## Methods

### Sample/study procedures

The study is a cross-sectional analysis of baseline data (*n* = 341) taken from the AL Amyloidosis Patient Health-Related Quality of Life Study (ClinicalTrials.gov; NCT02574676), an online noninterventional, longitudinal study of patients with AL amyloidosis. This study was approved by the New England Institutional Review Board. Recruitment took place between October and December 2015, with scripted messages posted on patient advocacy group Web sites and social media sites and in membership e-mails. These messages provided a hyperlink to an electronic informed consent form and screening questions. Potential participants were eligible to participate if they were ≥18 years of age, received a diagnosis of AL amyloidosis from a physician, and were willing and able to complete four online surveys over the course of 12 months. Those who met inclusion criteria were automatically directed to the baseline survey.

### Study measures

Several items capturing basic demographic, disease, and treatment characteristics were used to describe the sample. Dates of diagnosis and survey completion were used to calculate the time since each patient received the AL amyloidosis diagnosis. Specific organ involvement was measured with a six-item checklist of organs or systems commonly affected by AL amyloidosis, including heart (cardiac), kidney, liver, nervous system, and gastrointestinal system. Responses to these items were summed to generate a composite measure of the total number of organs affected. Hematologic response (HR), based on the assessments of serum-free light chains, is often used as a measure of treatment efficacy for AL amyloidosis [21]. In this study, a proxy measure of this clinical information was developed by asking patients to describe their most recent HR status in terms of the following: (1) no response to treatment, (2) partial HR or partial remission, (3) complete HR or complete remission, or (4) I do not know.

The SF-36v2 (with 4-week recall) was used to measure general HRQoL burden in eight dimensions of functional health and well-being: Physical Functioning (PF), Role-Physical (RP; role limitations due to physical problems), Bodily Pain (BP), General Health Perceptions (GH), Vitality (VT), Social Functioning (SF), Role-Emotional (RE; role limitations due to emotional problems), and Mental Health (MH). Item responses were used to calculate scale scores for each of the eight dimensions, and summary scores (Physical Component Summary [PCS] and Mental Component Summary [MCS]) were computed from weighted scores from the eight scales. All scores were calculated using a scoring algorithm that yields standardized T scores for a nationally representative sample of US adults [15]. Higher SF-36v2 scores represent better health. Previously reported minimal clinically important differences (MCIDs) for each of the eight scales, PCS, and MCS were used to interpret whether statistically significant differences were also clinically meaningful [15].

Two other survey items were used to describe burden in terms of current disease status or severity: a global assessment of functioning on a scale of 0 to 100 (higher scores mean better functioning) and the Patient Global Impression–Severity scale (PGI-S). The PGI-S was used to assess the severity of AL amyloidosis in the past month on a five-point scale (not severe at all, mild, moderate, severe, very severe) [22].

### Statistical analyses

Demographics, disease characteristics, and HRQoL were compared in stratified analyses of two clinically relevant subgroups: diagnosed <12 months ago versus diagnosed ≥12 months ago; cardiac involvement versus no cardiac involvement. Unadjusted differences between the subgroups were examined using chi-square and Wilcoxon-Mann–Whitney tests, as appropriate.

SF-36v2 scores for all patients and the key clinical subgroups were compared with those from the GP. Normative data were drawn from the QualityMetric 2009 Norming Study (*N* = 4040), a cross-sectional online study conducted between June and October 2009. The methodology for the GP has been described [15]. Participants were recruited from the Knowledge Panel®, a probability sample of US households [23], to complete an online survey that included the SF-36v2.

Data from the GP were adjusted to the age and gender distribution of the AL amyloidosis sample using separate least squares multiple regression models for each SF-36v2 scale and summary score. Analysis of variance methods were used to test for significant differences between scores obtained from the AL amyloidosis sample and the adjusted norms.

All analyses were performed using SAS version 9.2 (SAS Institute Inc, Cary, NC).

## Results

### Sample characteristics

Sample characteristics are reported in Table 1. The average age of the patients was 61 years. Slightly more women (52.9%) than men (47.1%) participated. Most patients were white (89.1%), well educated (61.2% had at least a 4-year college degree), and married (82.1%). Approximately 43% of patients were diagnosed >1 year after the onset of symptoms. Time since diagnosis varied from 1 month to 28 years (median, 3.5 years). Nearly 23% of the sample reported a history of multiple myeloma. There was a broad representation of affected organs; the largest percentage of patients had cardiac (52.2%) and/or kidney (62.8%) involvement. In approximately 46.0% of patients, three or more organs were affected by AL amyloidosis. At the time of data collection, most patients who had received at least one treatment series had experienced some type of response to treatment (39.3% partial HR, 43.6% complete HR).

**Table 1:** Demographic, Disease, and Treatment Characteristics of Study Participants

	Baseline sample	
	*N* = 341	
	*n*	%
Mean age, years (SD)	60.6 (10.2)	
Range, years (median)	23–85 (61)	
Gender (*n* = 340)^a^		
Male	160	47.1
Female	180	52.9
Race/ethnicity		
White	304	89.1
Other	37	10.9
Education (*n* = 322)^a^		
≤ High school diploma or GED®	26	8.1
Some college but no degree	50	15.5
A Associate’s degree or technical certificate	49	15.2
Bachelor’s degree	109	33.9
Graduate degree	88	27.3
Marital status (*n* = 330)^a^		
Married	271	82.1
Other	59	17.9
Employment status		
Currently employed for pay	115	38.3
US region of residence		
Northeast	68	19.9
Southeast	56	16.4
Midwest	54	15.8
Southwest	24	7.0
West	81	23.8
Other/unknown	58	17.0
Mean time since diagnosis, years (SD)	4.5 (4.0)	
Range (median)	1 month–28 years (3.5 years)	
Time between onset of symptoms and diagnosis		
Less than 6 months	96	28.2
Between 6 months and 1 year	97	28.4
Between 1 and 2 years	76	22.3
Between 2 and 3 years	31	9.1
More than 3 years	41	12.0
Number of doctors seen before diagnosis		
One	11	3.2
Two	57	16.7
Three	60	17.6
Four	68	19.9
Five or more	145	42.9
Organs/systems impacted^b^		
Heart (cardiac)	178	52.2
Kidney	214	62.8
Liver	49	14.4
Nervous system	126	37.0
Gastrointestinal	148	43.4
Other	117	34.3
Number of organs involved		
One	95	27.9
Two	89	26.1
Three or more	157	46.0
History of multiple myeloma	71	22.8
No. treatment series received		
None	20	5.9
One	70	20.5
Two	76	22.3
Three	61	17.9
Four	25	7.3
Five or more	89	26.1
Ever underwent stem cell transplantation	180	52.9
Most recent hematologic response status (*n* = 321)^c^		
No response to treatment	20	6.2
Partial hematologic response or partial remission	126	39.3
Complete hematologic response or complete remission	140	43.6
I do not know	35	10.9

GED, General Educational Development; SD, standard deviation

^a^Frequencies less than 341 are due to missing data; Percentages based on available data

^b^Multiple response options allowed

^c^Frequencies and percentages are based on the 321 patients who received at least one treatment series

Patient demographics did not differ significantly by time since diagnosis or cardiac involvement (data not shown). Patients diagnosed <12 months ago differed from those who received their diagnoses ≥12 months ago on several key aspects of disease and treatment (Table 2). Given their shorter duration of disease, greater proportions of patients diagnosed <12 months ago reported not having started treatment or having received only one treatment series to date. They were also less likely to have undergone SCT and less likely to have achieved complete HR than those with longer disease duration. Patients with cardiac involvement did not differ significantly from those without cardiac involvement by HR or history of SCT.

**Table 2:** Disease and Treatment Characteristics by Time since Diagnosis and Cardiac Involvement

	Time since diagnosis					Cardiac involvement				
	<12 months *n* = 52		≥12 months *n* = 289			No *n* = 163		Yes *n* = 178		
	*n*	%	*n*	%	*P* value	*n*	%	*n*	%	*P* value
No. organs involved					0.047					<0.001
One	20	38.5	75	26.0		71	43.6	24	13.5	
Two	16	30.8	73	25.3		42	25.8	47	26.4	
Three or more	16	30.8	141	48.8		50	30.7	107	60.1	
No. treatment series received										0.889
None	8	15.4	12	4.2	<0.001	12	7.4	8	4.5	
One	16	30.8	54	18.7		32	19.6	38	21.3	
Two	5	9.6	71	24.6		34	20.9	42	23.6	
Three	4	7.7	57	19.7		29	17.8	32	18.0	
Four	5	9.6	20	6.9		12	7.4	13	7.3	
Five or more	14	26.9	75	26.0		44	27.0	45	25.3	
Ever underwent stem cell transplantation (% yes)	5	9.8	175	60.6	<0.001	89	54.6	91	51.4	0.556
Most recent hematologic response status^a^					<0.008					0.135
No response to treatment	5	11.6	15	5.4		9	6.0	11	6.5	
Partial hematologic response or partial remission	19	44.2	107	38.5		62	41.1	64	37.7	
Complete hematologic response or complete remission	10	23.3	130	46.8		58	38.4	82	48.2	
I do not know	9	20.9	26	9.4		22	14.6	13	7.7	

^a^Frequencies and percentages are based on the 321 patients who received at least one treatment series

### The humanistic burden of AL amyloidosis

**Fig. 1:**
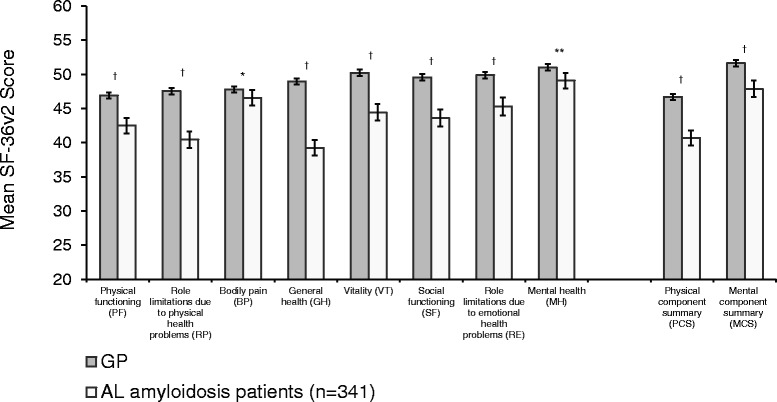
Mean SF-36v2 scores of patients with AL amyloidosis and of a general population. Error bars indicate 95% confidence intervals; GP adjusted to the age and gender distribution of sample of AL amyloidosis patients; GP sample size varied by scale/score: PF = 4034; RP = 4027; BP = 4027; GH = 4036; VT = 4028; SF = 4029; RE = 4026; MH = 4028; PCS = 4024 MCS = 4024. * GP > AL amyloidosis patients, *p* < 0.05. **GP > AL amyloidosis patients, *p* < 0.01. ^†^GP > AL amyloidosis patients, *p* < 0.001

Compared with age- and gender-adjusted GP norms, patients with AL amyloidosis had worse HRQoL, as demonstrated by their scores on all eight SF-36v2 scales and summary scores (*p* < 0.05 for all) (Fig. 1). All differences, with the exception of BP and MH, exceeded the established MCIDs, indicating that these deficits are clinically meaningful as well as statistically significant. The largest differences were related predominantly to physical health status. Compared with the GP, the largest observed differences were related to GH (Δ = 9.7; Cohen’s *d*, −0.65; *p* < 0.001) and RP (Δ = 7.1; Cohen’s *d*, −0.49; *p* < 0.001), with corresponding decrements in PCS (Δ = 5.9; Cohen’s *d*, −0.44; *p* < 0.001). Significant decrements were also seen for scales assessing mental health status, such as SF (Δ = 6.0; Cohen’s *d*, −0.40; *p* < 0.001) and VT (Δ = 5.8; Cohen’s *d*, −0.39; *p* < 0 .001), and a corresponding deficit was seen for the MCS (Δ = 3.7; Cohen’s *d*, −0.25; *p* < 0.001).

Overall SF-36v2 scores and other HRQoL measures are reported in Table 3. The average global assessment of functioning score was 70.2 (SD, 23.7). Based on responses to the PGI-S, slightly more than one-third of all patients described the severity of their condition as moderate to very severe (34.0%).

**Table 3:** Health-related quality of life among AL Amyloidosis patients (*n* = 341)

	AL amyloidosis patients *N* = 341	
	Mean (SD)	
SF-36v2 norm-based scales^a^		
Physical functioning (PF)	42.5 (10.6)	
Role physical (RP)	40.5 (11.3)	
Bodily pain (BP)	46.6 (10.6)	
General health (GH)	39.3 (10.6)	
Vitality (VT)	44.5 (11.1)	
Social functioning (SF)	43.6 (11.5)	
Role emotional (RE)	45.3 (12.3)	
Mental health (MH)	49.1 (10.6)	
SF-36v2 Summary Scores		
Physical Component Summary (PCS)	40.7 (10.3)	
Mental Component Summary (MCS)	47.9 (11.6)	
Global assessment of functioning	70.2 (23.7)	
Patient Global Impression–Severity Scale (PGI-S)	*n*	%
Not severe at all	133	39.0
Mild	92	27.0
Moderate	81	23.8
Severe	23	6.7
Very severe	12	3.5

^a^SF-36v2, SF-36v2 Health Survey

### The humanistic burden among patients with recently diagnosed AL amyloidosis

**Fig. 2:**
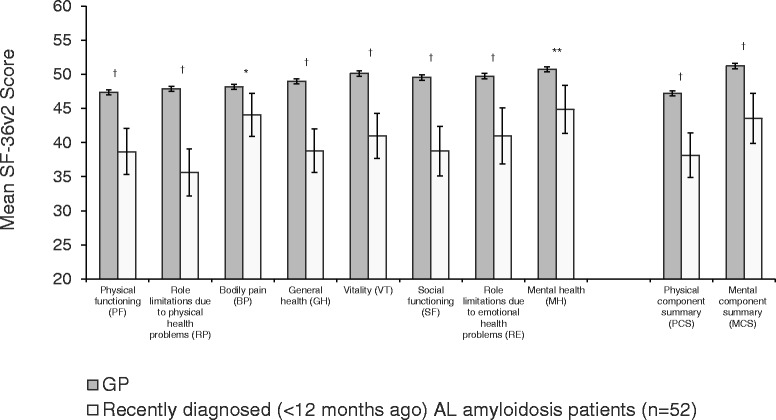
Mean SF-36v2 scores of patients with recently diagnosed AL amyloidosis and of a general population. Error bars indicate 95% confidence intervals; GP adjusted to the age and gender distribution of sample of AL amyloidosis patients; GP sample size varied by scale/score: PF = 4034; RP = 4027; BP = 4027; GH = 4036; VT = 4028; SF = 4029; RE = 4026; MH = 4028; PCS = 4024, MCS = 4024. *GP > AL amyloidosis patients, *p* < 0.05. **GP > AL amyloidosis patients, *p* < 0.01.^†^GP > AL amyloidosis patients, *p* < 0.001

Patients diagnosed <12 months earlier exhibited large decrements in all eight SF-36v2 scale scores, PCS, and MCS (*p* < 0.05 for all) compared with the GP (Fig. 2). Consistent with patterns observed in the overall sample, the burden among recently diagnosed patients was greater for physical health status than mental health status. The greatest HRQoL decrement among these patients was in RP (Δ = 12.3; Cohen’s *d*, −1.01; *p* < 0.001), with other large decrements in GH (Δ = 10.2; Cohen’s *d*, −0.81; *p* < 0.001) and SF (Δ = 10.8; Cohen’s *d*, −0.85; *p* < 0.001).

Deficits in HRQoL among AL amyloidosis patients with recent diagnoses often exceeded those among patients diagnosed ≥12 months ago (Table 4). Patients with recent diagnoses reported worse scores on PF, RP, VT, SF, RE, MH, and MCS than did those diagnosed ≥12 months ago (*p* < 0.05 for all). Furthermore, all differences exceeded the established MCIDs for these scales/scores. There were no significant differences for BP, GH, and PCS, though both groups of patients were severely impaired in these areas.

**Table 4:** Health-Related Quality of Life by Time since Diagnosis and Cardiac Involvement

	Time since diagnosis					Cardiac involvement				
	<12 months *n* = 52		≥12 months *n* = 289			No *n* = 163		Yes *n* = 178		
	Mean (SD)		Mean (SD)		*P* value	Mean (SD)		Mean (SD)		*P* value
SF-36v2 norm-based scales^a^										
Physical functioning (PF)	38.7 (12.4)		43.2 (10.2)		0.015	44.5 (10.9)		40.7 (10.0)		<0.001
Role physical (RP)	35.6 (12.7)		41.4 (10.9)		0.001	41.8 (11.7)		39.3 (10.9)		0.043
Bodily pain (BP)	44.0 (11.6)		47.0 (10.4)		0.099	47.2 (10.8)		46.1 (10.4)		0.257
General health (GH)	38.8 (11.7)		39.4 (10.3)		0.827	40.9 (10.5)		37.7 (10.4)		0.005
Vitality (VT)	40.9 (12.1)		45.1 (10.8)		0.027	44.9 (11.5)		44.1 (10.7)		0.530
Social functioning (SF)	38.7 (13.3)		44.5 (10.9)		0.003	43.8 (11.7)		43.5 (11.3)		0.786
Role emotional (RE)	41.0 (15.1)		46.1 (11.5)		0.038	45.3 (12.0)		45.3 (12.5)		0.900
Mental health (MH)	44.8 (13.0)		49.8 (10.0)		0.012	48.9 (10.8)		49.2 (10.5)		0.803
SF-36v2 Summary Scores										
P Physical Component Summary (PCS)	38.2 (11.9)		41.2 (9.9)		0.064	42.7 (10.6)		38.9 (9.6)		<0.001
Mental Component Summary (MCS)	43.5 (13.5)		48.7 (11.1)		0.012	47.2 (11.9)		48.5 (11.4)		0.348
Global assessment of functioning	59.1 (31.2)		72.2 (21.6)		0.015	73.1 (23.5)		67.5 (23.7)		0.013
Patient Global Impression–Severity Scale (PGI-S)	*n*	%	*n*	%		*n*	%	*n*	%	
					<0.001					<0.001
Not severe at all	11	21.2	122	42.2		84	51.5	49	27.5	
Mild	12	23.1	80	27.7		40	24.5	52	29.2	
Moderate	15	28.8	66	22.8		26	16.0	55	30.9	
Severe	9	17.3	14	4.8		8	4.9	15	8.4	
Very severe	5	9.6	7	2.4		5	3.1	7	3.9	

^a^SF-36v2, SF-36v2 Health Survey

Responses to the global assessment of functioning and the PGI-S reinforced the finding of added burden among patients with recent diagnoses (Table 4). The mean global assessment of functioning score was approximately 22% greater for those with diagnoses made ≥12 months ago than for those with more recent diagnoses (72.2 vs 59.1, respectively; *p* < 0.05), indicating better functioning among those whose disease was diagnosed for a longer period of time. A greater proportion of patients with recent diagnoses described the severity of their disease as moderate to very severe (55.7%) than did patients diagnosed ≥12 months ago (30.0%) (*p* < 0.001).

### The humanistic burden among AL amyloidosis patients with cardiac involvement

**Fig. 3:**
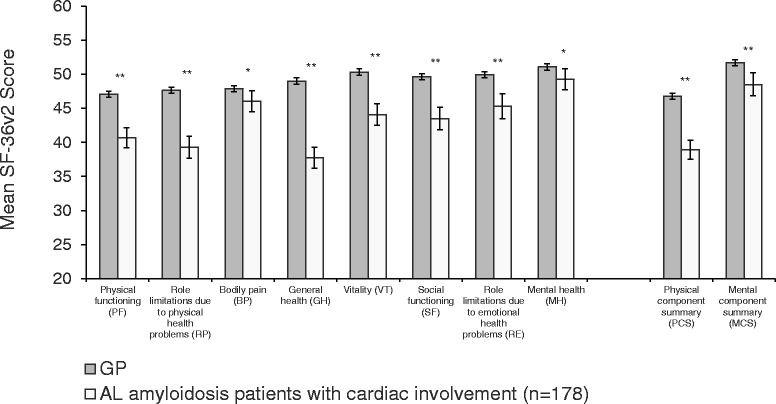
Mean SF-36v2 scores of patients with AL amyloidosis and cardiac involvement and of a general population. Error bars indicate 95% confidence intervals; GP adjusted to the age and gender distribution of sample of AL amyloidosis patients; GP sample size varied by scale/score: PF = 4034; RP = 4027; BP = 4027; GH = 4036; VT = 4028; SF = 4029; RE = 4026; MH = 4028; PCS = 4024, MCS = 4024. *GP > AL amyloidosis patients, *p* < 0.05. **GP > AL amyloidosis patients, *p* < 0.001

Compared with the GP, patients with cardiac involvement also reported large decrements on all eight SF-36v2 scales and summary scores (*p* < 0.05 for all) (Fig. 3). With the exception of BP and MH, all differences exceeded established MCIDs. Similar to the finding in the overall sample, the largest deficits were for scales that contribute to physical health status (ie, PF, RP, and GH). Similarly, AL amyloidosis patients with cardiac involvement had significantly lower PCS scores than the adjusted GP (*p* < 0 .001).

Results indicate that AL amyloidosis with cardiac involvement is associated with greater physical impairment than with non-cardiac organ involvement (Table 4). Mean SF-36v2 scores for patients with cardiac involvement were significantly lower for patients without cardiac involvement for three of the four physical scales (PF, RP, and GH; *p* < 0.05 for all) and subsequently for PCS (*p* < 0.001). No significant differences were observed for MH scores or the MCS.

Responses on the PGI-S and global assessment of functioning items differed by presence of cardiac involvement (Table 4). Patients with cardiac involvement were half as likely as those without cardiac involvement to classify their condition as “not severe at all” (27.5% vs 51.5%, respectively). A significantly greater proportion of patients with cardiac involvement than without it described the severity of their disease as moderate to very severe (43.2% vs 24.0%, respectively) (*p* < 0.001).

## Discussion

These results indicate that AL amyloidosis patients have broad HRQoL deficits relative to a general population. Decrements in physical and mental functioning were statistically significant and often exceeded thresholds for clinically meaningful differences. The largest effects were observed in aspects related to physical functioning and general well-being. For instance, GH and RP were among the greatest deficits observed overall and in each key subgroup.

As expected, there was greater impairment in patients with recent diagnoses and those with cardiac involvement. Cardiac involvement in AL amyloidosis can lead to complications such as cardiomyopathy and heart failure. Although there are no other known studies that report HRQoL specifically in AL amyloidosis patients with cardiac involvement, our findings are congruent with assessments in populations with non-amyloidosis cardiomyopathy and heart failure [24].

This is the first study to characterize HRQoL in a community-based sample of AL amyloidosis patients and to further document HRQoL specifically in cardiac AL amyloidosis patients. Previous HRQoL-related studies in AL amyloidosis used older versions of the SF-36, relied on clinic-based samples, and predated many of the newer drugs used to treat AL amyloidosis [13, 14]. By partnering with patient advocacy groups, we were able to overcome some of the challenges of sample accrual and data collection often experienced in studies of rare diseases. Since recruitment occurred outside of the clinic setting, we were able to obtain a diverse sample of patients that included patients with recent diagnoses and long-term survivors. Rather than having patients complete a time-intensive survey in a clinic setting, the centralized, online mode of data collection used in this study allowed patients to complete the survey on their own time and from any location, which facilitated participation.

Despite the benefits of our recruitment and data collection strategies, several limitations are worth noting. All measures in this study relied on self-report. Although some measures were meant to be subjective (ie, the PGI-S), other measures of disease severity were meant to be proxies for objective measures, such as HR or cardiac involvement. These proxy measures may have been affected by measurement error. HR, in particular, relies on patients’ recall and understanding of information relayed to them by their clinicians. A notable proportion of the sample (11%) acknowledged their uncertainty of their current HR status by endorsing the ‘I do not know’ category.

Our sample may represent a healthier subset of AL amyloidosis patients. First, despite advances in the treatment of patients with AL amyloidosis, the prevalence of sudden death within 90 days of diagnosis remains around 25–30%, whereas patients who survive >12 months have a better prognosis [25–27]. Here, 85% of patients in this sample received their diagnoses ≥12 months ago, and the median time since diagnosis within this sample was 3.5 years. This is typical of cross-sectional studies in which short-term survivors are often underrepresented among prevalent cases [28].

Second, only the healthiest AL amyloidosis patients (<20% of all patients) are typically eligible to undergo SCT [29], but the lifetime prevalence of SCT in this sample was 53%. Finally, 41% of the sample reported current complete remission or HR, indicating that a substantial proportion of this sample responded favorably to treatment.

Despite the likelihood that this sample might have been disproportionately healthier than the broader population of AL amyloidosis patients, the demographic characteristics of our study population mirror those previously described in the literature, including those of patients with newly diagnosed AL amyloidosis and long-term survivors with a range of organ involvement [3, 14]. However, given the likelihood that our sample represented healthier patients, it is probable that the HRQoL in patients with AL amyloidosis is actually lower.

These findings may help clinicians understand how their patients are impacted by AL amyloidosis. Understanding the burden of AL amyloidosis can help physicians identify ancillary treatments and services that may ease patients’ disease burden and ultimately improve their HRQoL. Subsequently, this may improve the doctor-patient relationship through a patient-centered approach.

Furthermore, these findings may interest regulatory groups, such as the FDA and the EMA. FDA’s Guidance for Industry in Rare Diseases specifically notes that rare diseases are highly diverse and that, due to the small number of patients affected and the clinical expertise dispersed among a small number of treatment centers, the natural history of rare diseases is often poorly described, as are “patient reports of function and feeling” [30]. The FDA notes that drug developers should conduct observational studies to facilitate understanding of the population and unmet need, to define the disease population and subtypes, and to design appropriate clinical trials with suitable outcome measures that are sensitive to change in disease status and that are reliable and valid for the intended use.

## Conclusions

Overall these data demonstrated significant deficits in HRQoL among AL amyloidosis patients and an additional burden among clinically relevant subgroups of patients. Further, this study indicates that the SF-36v2 is sensitive to variations in HRQoL across groups known to differ in disease severity. The study is continuing to collect data to evaluate the change in HRQoL over time and in other subgroups. Given the importance of providing patient-centered care and including the patient’s voice in research, these findings are relevant to a variety of stakeholders, among them physicians, patients, payors, and regulatory groups.

## Abbreviations

AL: Amyloid light chain
BP: Bodily pain
EMA: European medicines agency
FDA: United States food and drug administration
GED: General educational development
GH: General health
GP: General population
HR: Hematologic response
HRQoL: Health-related quality of life
ICF: Informed consent form
MCID: Minimal clinically important difference
MCS: Mental component summary
MH: Mental health
PCS: Physical component summary
PF: Physical functioning
PGI-S: Patient global impression–severity scale
RE: Role emotional
RP: Role physical
SCT: Stem cell transplantation
SD: Standard deviation
SF: Social functioning
SF-36v2: SF-36v2 health survey®
VT: Vitality
